# Traditional, cyberbullying, and suicidal behaviors in Argentinian adolescents: the protective role of school, parental, and peer connectedness

**DOI:** 10.3389/fpsyt.2024.1351629

**Published:** 2024-03-04

**Authors:** Omid Dadras, Naoki Takashi

**Affiliations:** ^1^ Department of Global Public Health and Primary Care, University of Bergen, Bergen, Norway; ^2^ Bergen Addiction Research, Department of Addiction Medicine, Haukeland University Hospital, Bergen, Norway; ^3^ Department of Health Economics, Center for Gerontology and Social Science, National Center for Geriatrics and Gerontology, Aichi, Japan; ^4^ Department of Health Informatics, School of Public Health, Graduate School of Medicine, Kyoto University, Kyoto, Japan

**Keywords:** adolescents, bullying, cyberbullying, suicide, Argentina

## Abstract

**Introduction:**

Bullying, both in person and online, is a significant risk factor for a range of negative outcomes including suicidal behaviors among adolescents and it is crucial to explore the protective effects of parental, school, and peer connectedness on suicidal behaviors among victims.

**Methods:**

This study is a secondary analysis of the Argentina Global School-based Student Health Survey (GSHS 2018). Logistic regression analysis, adjusting for age and sex, determines the likelihood of suicidal thoughts and attempts among bullying victims. To explore the modifying effect of school, parental, and peer connectedness on the association between bullying and suicide behaviors, the interaction term was included. Sampling design and weights were applied in all analyses in STATA 17.

**Results:**

The study included 56,783 students in grades 8-12, with over half being female. Adolescents aged 14-15 exhibited the highest prevalence of bullying, cyberbullying, suicidal thoughts, and attempts, with females displaying a higher prevalence in all measured categories. The study found that adolescents who reported being bullied or cyberbullied demonstrated a significantly higher likelihood of experiencing suicidal thoughts and attempting suicide. Furthermore, protective factors such as school, parental, and peer connectedness were found to play a critical role in mitigating the adverse impacts of bullying and cyberbullying on suicidal thoughts and attempts.

**Conclusion:**

The findings underscore the critical prevalence of both bullying and cyberbullying among school-going Argentinian adolescents and their profound association with suicidal behaviors. The study emphasizes the importance of supportive family environments and peer and school connectedness in mitigating the negative effects of bullying and cyberbullying on mental health and suicide risk among adolescents.

## Introduction

Bullying is a pervasive issue in schools, characterized by repeated aggressive behavior and an imbalance of power (1). This is considered cyberbullying if it involves using information and communication technologies (ICTs) to harm others. Cyberbullying occurs most frequently through email and social networking sites, with mobile phones being the primary method in some countries (2). Bullying, both in person and online, is a significant risk factor for a range of negative outcomes including mental health issues and suicidal behaviors among school-going adolescents, with a higher prevalence in Latin America and other low- and middle-income countries (3–5). Approximately 10-20% of youth are bullied by their peers, and 5-15% engage in bullying behavior (6). The association between bullying and suicide risk is particularly concerning, with studies indicating that any involvement in bullying increases the likelihood of suicidal thoughts and behaviors (5, 6). This correlation is particularly concerning among school-going adolescents in Argentina with approximately 22% of students aged 13-17 years experiencing bullying victimization and having suicidal thoughts in the past 12 months according to the Global School-based Student Health Survey 2018 (GSHS 2018) (7).

Bullied children and adolescents may exhibit psychosomatic symptoms such as headaches and musculoskeletal pains, which can be indicative of deeper psychological distress and potential suicidal behaviors. Research has shown that bullying can cause an emotional toll on its victims, leading to physical manifestations of mental anguish, including symptoms like unusual headaches, loss of appetite, sleeping problems, and abdominal pain (8–11). These psychosomatic symptoms serve as important indicators of the psychological impact of bullying, and recognizing this connection is crucial for early diagnosis and intervention to prevent suicidal ideation and attempts among bullied youths (10). The impact of bullying on mental health can be long-lasting, leading to feelings of rejection, exclusion, isolation, low self-esteem, depression, and anxiety (12). Therefore, it is essential to address bullying and its associated psychological and physical consequences to ensure the well-being of children and adolescents.

In addition to the detrimental effects of bullying victimization, the protective role of connectedness to family, school, peers, and community has been highlighted in mitigating the risk of adverse outcomes among adolescents (13, 14). It has been found that family and school connectedness was associated with lower levels of depressive symptoms, suicidal ideation, and conduct problems, as well as higher self-esteem and more adaptive use of free time (13, 14). Additionally, the importance of social support, particularly from family, in promoting good mental health and protecting bullied adolescents from poor academic achievement has been documented (15). Research also underscored the significance of caring and connectedness, particularly a sense of connectedness to family and school, in reducing high-risk behaviors among youth (16) and those who felt more connected to parents and school reported lower levels of depressive symptoms, suicidal ideation, and non-suicidal self-injury (13).

Given the significance of these issues, it is crucial to explore the protective effects of parental, school, and peer connectedness on suicidal behaviors among bullying victims among Argentinian adolescents. In particular, this research focuses on whether Argentinian students who have been the victim of either traditional or cyberbullying experience more suicidal thoughts and attempts and how the suggested protective factors including parental understanding, school connectedness, and having close friends could modify this relationship. By understanding the effect of these potential protective factors, strategies, and interventions can be formulated to reduce the prevalence of bullying and suicidal behaviors among adolescents in Argentina.

## Methods

### Study setting

This cross-sectional study used data from the 2018 Argentina Global School-based Student Health Survey (GSHS), a nationally representative school-based survey among students in 8-12^th^ grades in Argentina. The GSHS uses self-administered questionnaires to collect information on health behavior and protective factors among a nationally representative sample of school-going adolescents in different countries.

### Participants

Argentina GSHS 2018 used a two-stage cluster sampling to recruit a representative sample of Argentinian students in the 8^th^ grade (primary/polymodal schools) to 12 fifth-grade (polymodal schools). In the first stage, schools were selected proportionally to the size of the school. In the second stage, classes were randomly selected and all students in those classes were invited to participate in the survey and complete a self-administered questionnaire in Spanish [11]. The students were informed about the study objectives their right to withdraw from the study and voluntary nature of their participation as well as the anonymity and confidentiality of collected data. The response rate was 86% for schools and 74% for students with an overall response rate of 63%. A total sample of 56,981 adolescents in 8-12^th^ grades participated in the survey [11] and was included in this study to assess the associations between suicidal and bullying behaviors and the protective effect of school, parental, and peer connectedness.

### Outcome variable

The outcomes of interest in this study were suicide ideation and attempt. Suicide thoughts were assessed by the question “During the past 12 months have you ever seriously considered attempting suicide?”. The suicide attempt was assessed by the question “During the past 12 months, how many times did you attempt suicide?” The answers to both questions were coded as 1 “yes” and 0 “no”.

### Independent variables

The exposure variables of interest in this study were traditional bullying and cyberbullying. Traditional bullying was measured by asking the questions “During the past 12 months, have you ever been bullied on school property” and “During the past 12 months, have you ever been bullied when you were not on school property?”. The responses to this question were combined to construct a binary variable for bullying with alternative categories of 1 “yes” and 0 “no”. Cyberbullying was measured by asking the question “During the past 12 months, have you ever been cyberbullied? (Count being bullied through texting, Instagram, Snapchat, Facebook, Whatsapp, Edmodo, Messenger, or other social media.)” and the response was binary (1 “yes” and 0 “no”).

The protective variable of interest was school connectedness which was assessed by asking the question “During the past 30 days, how many days did you miss classes or school without permission?”, a desirable school connectedness was considered no missing classes during past 30 days and thus, the responses were coded as 1 “yes” and 0 “no”. The parental-child (parental) connectedness was assessed by the question “During the past 30 days, how often did your parents or guardians understand your problems and worries?” and the responses were categorized as always/often “1” and never/rarely/sometimes “0”. The peer connectedness was assessed by asking the question “How many close friends do you have?” having even one close friend was considered desirable and the responses were coded as 1 “yes” and 0 “no”.

### Covariates

Age and sex are the most important influencing factors on both traditional and cyberbullying (17–19) as well as suicidal behaviors and therefore, to measure the modifying impact of the protective factors in this study, we have adjusted all the models for the confounding effect of these two variables (20). Additionally, we have also included grades (8-12^th^) as a covariate, however, due to collinearity between this variable and age, it was not included in the multivariate analyses.

### Statistical analysis

Descriptive statistics were employed to describe the sample demography and the distribution of outcome (suicide thoughts and attempts) and exposure variables (traditional bullying, cyberbullying) across demographic characteristics (age, sex, grade) among Argentinian adolescents in grades 8–12^th^. The chi-square test examined the association of exposure and outcome variables with demographic characteristics (**Table 1**). Logistic regression analysis, adjusting for age and sex, determines the likelihood of suicidal thoughts and attempts among bullying victims. To explore the modifying effect of school, parental, and peer connectedness on the association between bullying and suicide behaviors, the interaction term was included in multivariate analyses and the marginal effect was examined reporting the p-value for the interaction term. To specify the protective effect, the population was restricted to bullying victims, and the odds of suicide thoughts and attempts were examined across the categories of school, parental, and peer connectedness, and the results were reported as odds ratio (OR) and 95% confidence interval (95%CI). Due to the complex sampling design in Argentina GSHS 2018, sampling design and weights were defined and applied in all analyses in STATA 17. The statistical significance level was set at p< 0.05.

**Table 1:** The prevalence of suicidal and bullying behaviors among school-going Argentinian adolescents; by age, sex, and grade.

	Total	Bullying	Cyberbullying	Suicide thoughts	Suicide attempts
	n (%) ^1^	n (%) ^1^	n (%) ^1^	n (%) ^1^	n (%)^1^
Age					
<14	10,767 (21.04)	3,464 (21.28)	1,922 (17.15)	2,086 (19.89)	1,607 (20.90)
14-15	25,758 (47.02)	8,288 (46.92)	5,635 (48.53)	5,632 (48.25)	4.017 (48.31)
≥16	20,348 (31.94)	6,434 (31.80)	4,615 (34.33)	4,228 (31.86)	2,863 (30.78)
*p-value ^2^*		0.854	<0.001	0.188	0.499
Sex					
Male	27,083 (48.01)	7,887 (44.07)	4,204 (36.29)	3,682 (30.84)	2,831 (34.06)
Female	29,362 (51.99)	10,134 (56.23)	7,873 (63,71)	8,149 (69.16)	5,543 (65.94)
*p-value^2^*		<0.001	<0.001	<0.001	<0.001
Grade					
8th	8,581 (14.75)	2,708 (15.07)	1,603 (12.90)	1,701 (14.12)	1,429 (17.20)
9th	11,913 (26.85)	3,948 (27.58)	2,479 (24.61)	2,528 (26.53)	1,895 (26.19)
10th	12,448 (23.27)	4,000 (23.03)	2,770 (24.88)	2,795 (25.34)	1,876 (24.83)
11th	13,275 (18.94)	4.221 (18.99)	2,976 (20.63)	2,850 (19.61)	1,925 (17.74)
12th	9,703 (16.20)	2,975 (15.33)	2,168 (16.98)	1,893 (14.57)	1,215 (14.04)
*p-value^2^*		0.411	<0.001	0.002	0.006

^1^Weighted proportion. ^2^Chi-square test p-value.

## Results

### Sample characteristics

A total of 56,783 students in grades 8-12^th^ included in the present study. More than half were female (51.99%). The majority were in 9^th^ (26.85%) and 10^th^ (23.27%) grades.

### Suicidal and bullying behaviors among Argentinian adolescents, by age, sex, and grade

**Table 1** illustrates the prevalence of suicidal and bullying behaviors among Argentinian adolescents in grades 8-12th. No significant differences were observed in bullying prevalence across age groups (p = 0.854). However, significant differences were found in cyberbullying (p < 0.001) and suicide thoughts (p = 0.188) among different age categories. Adolescents aged 14-15 exhibited the highest prevalence of bullying (46.92%), cyberbullying (48.53%), suicide thoughts (48.25%), and suicide attempts (48.31%); followed by those aged ≥16 years with the lowest prevalence among those aged <14 years old. Females displayed higher prevalence in all measured categories, notably in cyberbullying (63.71%), suicide thoughts (69.16%), and suicide attempts (65.94%). Significant differences were observed between sexes across all measured categories (p < 0.001). Middle grades (9^th^-11^th^) demonstrated a slightly higher prevalence of bullying and suicide behaviors. Significant differences were observed in cyberbullying (p < 0.001), suicide thoughts (p = 0.002), and suicide attempts (p = 0.006) among different grades.

### Relationship between bullying and suicidal behaviors among Argentinian adolescents in grades 8-12^th^

Adolescents who reported being bullied demonstrated significantly higher likelihoods of experiencing suicidal thoughts (PRR: 2.68, 95% CI: 2.50-2.87) and attempting suicide (PRR: 2.35, 95% CI: 2.10-2.63) compared to those who were not bullied. As **Table 2** illustrates, all interactions between bullying and protective factors were statistically significant (p < 0.001), highlighting the significant moderating roles of school, parental, and peer connectedness in attenuating the impact of bullying on suicidal behaviors among adolescents. Among bullying victims, having favorable school connectedness significantly reduced the likelihood of suicide thoughts (PRR: 0.70, 95% CI: 0.60-0.82) and suicide attempts (PRR: 0.62, 95% CI: 0.54-0.71) compared to victims without such connectedness. Bullying victims with strong parental connectedness exhibited notably lower likelihoods of suicide thoughts (PRR: 0.39, 95% CI: 0.33-0.46) and suicide attempts (PRR: 0.46, 95% CI: 0.40-0.53) compared to victims lacking such connections with parents. Bullying victims who reported having good peer connectedness showed reduced likelihoods of suicidal thoughts (PRR: 0.45, 95% CI: 0.37-0.55) and suicide attempts (PRR: 0.47, 95% CI: 0.38-0.59) compared to those without close peer connections.

**Table 2:** The relationship between bullying and behaviors among school-going Argentinian adolescents in grades 8-12^th^, GSHS 2018.

	Suicide thoughts		Suicide attempts	
	PRR (95%CI)^1^	*p-value^2^*	PRR (95%CI)^1^	*p-value^2^*
Bullying				
Yes	2.68 (2.50, 2.87)		2.35 (2.10, 2.63)	
No	Reference		Reference	
*Bullying#School connectedness*		*<0.001*		*<0.001*
Yes#Yes	0.70 (0.60, 0.82)^3^		0.62 (0.54, 0.71)^3^	
Yes#No	Reference		Reference	
*Bullying#Parental connectedness*		*<0.001*		*<0.001*
Yes#Yes	0.39 (0.33, 0.46)^3^		0.46 (0.40, 0.53)^3^	
Yes#No	Reference		Reference	
*Bullying#Peer connectedness*		*<0.001*		*<0.001*
Yes#Yes	0.45 (0.37, 0.55)^3^		0.47 (0.38, 0.59)^3^	
Yes#No	Reference		Reference	

^1^Adjusted for age and sex. ^2^p-value for the interaction effect. ^3^Restricted to bullying victims.

### Relationship between cyberbullying and suicidal behaviors among Argentinian adolescents in grades 8-12^th^

Adolescents who experienced cyberbullying exhibited significantly higher likelihoods of reporting suicidal thoughts (PRR: 2.81, 95% CI: 2.63-3.99) and attempting suicide (PRR: 2.58, 95% CI: 2.36-2.83) compared to those who did not report cyberbullying incidents. As **Table 3** indicates, all interactions between cyberbullying and protective factors were statistically significant (p < 0.001), emphasizing the significant moderating roles of school, parental, and peer connectedness in mitigating the impact of cyberbullying on suicidal behaviors among adolescents. Among cyberbullying victims, favorable school connectedness significantly reduced the likelihood of suicidal thoughts (PRR: 0.75, 95% CI: 0.60-0.93) and suicide attempts (PRR: 0.67, 95% CI: 0.54-0.84) compared to victims without such connectedness. Adolescents experiencing cyberbullying but having strong parental connectedness exhibited notably lower likelihoods of suicide thoughts (PRR: 0.42, 95% CI: 0.36-0.49) and suicide attempts (PRR: 0.49, 95% CI: 0.40-0.61) compared to victims lacking such connections with parents. Cyberbullying victims who reported having good peer connectedness showed reduced likelihoods of suicide thoughts (PRR: 0.48, 95% CI: 0.38-0.61) and suicide attempts (PRR: 0.48, 95% CI: 0.36-0.64) compared to those without close peer connections.

**Table 3:** The relationship between cyberbullying and behaviors among school-going Argentinian adolescents in grades 8-12^th^, GSHS 2018.

	Suicide thoughts		Suicide attempts	
	PRR (95%CI)^1^	*p-value^2^*	PRR (95%CI)^1^	*p-value^2^*
Cyberbullying				
Yes	2.81 (2.63, 3.99)		2.58 (2.36-2.83)	
No	Reference		Reference	
*Cyberbullying#School connectedness*		*<0.001*		*<0.001*
Yes#Yes	0.75 (0.60, 0.93)^3^		0.67 (0.54, 0.84)^3^	
Yes#No	Reference		Reference	
*Cyberbullying#Parental connectedness*		*<0.001*		*<0.001*
Yes#Yes	0.42 (0.36, 0.49)^3^		0.49 (0.40, 0.61)^3^	
Yes#No	Reference		Reference	
*Cyberbullying#Peer connectedness*		*<0.001*		*<0.001*
Yes#Yes	0.48 (0.38, 0.61)^3^		0.48 (0.36, 0.64)^3^	
Yes#No	Reference		Reference	

^1^Adjusted for age and sex. ^2^p-value for the interaction effect. ^3^Restricted to bullying victims.

## Discussion

The findings of this study underscore the critical prevalence of both bullying and cyberbullying among Argentinian adolescents and their profound association with suicidal behaviors, highlighting several noteworthy points. Adolescents aged 14-15 experienced the highest prevalence of bullying (46.92%), cyberbullying (48.53%), suicide thoughts (48.25%), and suicide attempts (48,31%); followed by those older than 16 years old. Studies have highlighted mid-adolescence (around 14-15 years) as a critical period where bullying, cyberbullying, and suicidal tendencies tend to peak. This peak in mid-adolescence aligns with the developmental phase where physical and psychological changes are most pronounced (21, 22) and often marks increased vulnerability to social stressors and peer influences, contributing to heightened prevalence (23, 24), with females being more affected than males (25). This vulnerability is exacerbated by the developmental factors of adolescence, such as heightened emotionality and risk-taking behavior (26). These findings underscore the need for targeted interventions and support for adolescents during this pivotal developmental phase. The exact reasons for gender differences remain unclear, but some theories suggest they might stem from variations in socialization, coping mechanisms, and the nature of bullying experienced by different genders (27).

The investigation into the relationship between bullying/cyberbullying and suicidal behaviors among Argentinian adolescents emphasized significantly elevated risks among victims. This is in line with previous studies indicating a greater risk of self-harm and suicidal behaviors among bullying and cyberbullying victims (24, 28, 29). This has been attributed to factors such as hopelessness, lowered self-esteem, psychological insecurity, increased fears of loneliness, emotional intelligence, and depressive symptoms (24, 30). These findings have significant implications for policies and interventions aimed at addressing bullying and cyberbullying among adolescents. It is crucial to recognize the heightened risk of suicidal behaviors among victims of bullying and cyberbullying and to implement targeted interventions to address these issues. This may involve the development and implementation of comprehensive anti-bullying programs in schools, as well as mental health support services for victims of bullying and cyberbullying. It is also important to recognize that adolescents subjected to bullying often have pre-existing personal issues that exacerbate their vulnerability, suggesting that interventions should focus on addressing these primary personal issues alongside bullying (31–33). These issues can range from poor parental support and monitoring to academic and social difficulties. The role of schools in identifying and supporting these adolescents is crucial, considering that both bullies and victims often face family and personal challenges (31). Therefore, a comprehensive approach to addressing bullying should also take into account the underlying personal fragilities that may contribute to both bullying and poor social connections (31, 32).

The findings of the present study also showed that protective factors such as school connectedness can have a critical role in mitigating the adverse impacts of bullying and cyberbullying on suicidal thoughts and attempts among school-going Argentinian adolescents. Research consistently demonstrates the protective role of school connectedness in mitigating the adverse impacts of bullying and cyberbullying on adolescent mental health (20, 34). Evidence have shown that youth who feel connected to their school are less likely to experience poor mental health, sexual health risks, substance use, and violence (35). Higher levels of school connectedness are associated with lower levels of suicidal behavior and depression, and with higher levels of self-esteem. This is particularly true for victims of cyberbullying, suggesting that school connectedness can buffer the negative effects of cyberbullying on mental health (34). A similar study among Korean adolescents found that school connectedness moderated the relationship between cyberbullying victimization and suicidal ideation, regardless of SES. These findings underscore the importance of school connectedness in protecting adolescents from the harmful effects of bullying and cyberbullying.

Another important finding of the present study was the moderating effect of parent-child connectedness on the association between bullying and suicidal behaviors among school-going Argentinian adolescents. A study on family factors related to suicidal behavior in adolescents found that insecure attachment and rigid or negligent relationships between parents and children were associated with bullying and cyberbullying, which in turn were risk factors for suicidal behavior (36). Insecure attachment, resulting from parenting that is inattentive and unsupportive, can lead to children perceiving themselves as unlovable and others as harsh, and it has been correlated with a lack of interpersonal sensitivity and heightened aggression (36, 37). Additionally, parental styles of rejection and indifference and the non-disclosure of bullying and suicidal ideation. This point further underscores the significance of parental involvement in mitigating the risks associated with bullying (38). Another study suggested that secure attachment to parents can moderate the association between sibling bullying and depression/suicidal ideation among children and adolescents, leading to a weaker association between sibling bullying involvement and depression/suicidal ideation among children with secure attachments with their parents (39). Additionally, research performed in different countries has indicated that bullying at school is associated with psychological distress and suicidal behaviors, highlighting the importance of parent-adolescent bonding in mitigating these negative outcomes (40–42). These findings underscore the crucial role of parent-child connectedness in influencing the relationship between bullying and suicidal behaviors, emphasizing the need for supportive family environments to promote adolescents’ mental health. This can be facilitated through implementing programs that aim to improve both the parental and the children behavior. This could include parenting skills training, stress management, and strategies for effective family communication (43).

Having close friends, translated into peer connectedness in the present study, also appeared to moderate the effect of bullying victimization on the likelihood of suicidal thoughts and attempts. A study found that adolescents who reported greater improvements in peer connectedness were half as likely to attempt suicide during 12 months (44). Social connectedness, particularly with close friends, can play a protective role in mitigating the negative effects of bullying victimization on suicide risk (14, 45, 46). These findings highlight the importance of peer and school connectedness in mitigating the negative effects of bullying victimization on mental health and suicide risk among adolescents. Thus, policies and interventions should focus on promoting school, parental, and social connectedness among adolescents to mitigate the negative impact of bullying and cyberbullying on mental health.

### Limitations

The study utilized data from the 2018 Argentina Global School-based Student Health Survey (GSHS), which employed self-administered questionnaires to collect information on health behavior and protective factors among a nationally representative sample of school-going adolescents in Argentina. However, the response rate was 63%, which may introduce non-response bias and affect the generalizability of the findings. Nonetheless, the large sample size of the study compensates for that, thereby ensuring the robustness and broader applicability of the results. The cross-sectional nature of the study design limits the ability to establish causal relationships between the variables of interest. Additionally, the study relied on self-reported measures for outcomes such as suicide ideation and attempt, as well as exposure variables like traditional bullying and cyberbullying which may be subject to recall bias and social desirability bias, potentially impacting the accuracy of the results. While the study adjusted for age and sex, other potential confounding variables such as socioeconomic status, mental health history, and family dynamics were not included. Failure to account for these factors may limit the ability to draw robust conclusions about the associations observed.

## Conclusion

In conclusion, the findings of this study underscore the critical prevalence of both bullying and cyberbullying among school-going Argentinian adolescents in grades 8-12 and their profound association with suicidal behaviors. The study highlights the need for targeted interventions to address these issues, including the development and implementation of comprehensive anti-bullying programs in schools, and mental health support services for victims of bullying and cyberbullying. The study also emphasizes the importance of supportive family environments and peer and school connectedness in mitigating the negative effects of bullying and cyberbullying on mental health and suicide risk among adolescents. Given the gravity of the issue, in 2022, for the first time, the Argentinian government introduced a national strategy aimed at preventing, addressing, and eradicating bullying and cyberbullying in children and youth. This initiative is outlined in Bill No. 3038-D/2022, which focuses on creating a comprehensive approach to combat these issues in educational institutions in the country. The effect of this program in reducing bullying, however, should be explored in future studies.

## Data availability statement

Publicly available datasets were analyzed in this study. This data can be found here: https://extranet.who.int/ncdsmicrodata/index.php/catalog.

## Ethics statement

This was a secondary analysis of the Argentina Global School-Based Student Health Survey conducted in 2018 (GSHS 2018). The GSHS protocol received approval and guidance from Argentina’s Ministry of Education and the Ministry of Public Health and written informed consent was obtained from the participants or their guardians before the survey.

## Author contributions

OD: Conceptualization, Data curation, Formal analysis, Methodology, Software, Visualization, Writing – original draft, Writing – review & editing. NT: Investigation, Methodology, Validation, Visualization, Writing – original draft, Writing – review & editing.
